# Alisol B 23-Acetate Ameliorates Lipopolysaccharide-Induced Intestinal Barrier Dysfunction by Inhibiting TLR4-NOX1/ROS Signaling Pathway in Caco-2 Cells

**DOI:** 10.3389/fphar.2022.911196

**Published:** 2022-06-14

**Authors:** Fan Xia, Yuxin Li, Lijun Deng, Ruxia Ren, Bingchen Ge, Ziqiong Liao, Shijian Xiang, Benjie Zhou

**Affiliations:** ^1^ Department of Pharmacy, The Seventh Affiliated Hospital of Sun Yat-sen University, Shenzhen, China; ^2^ Department of Pharmacology, Guangdong Medical University, Zhanjiang, China

**Keywords:** alisol B 23-acetate, intestinal barrier, tight junctions (TJs), NADPH oxidase-1 (NOX-1), toll-like receptor-4 (TLR4)

## Abstract

Alisol B 23-Acetate (AB23A) is a naturally occurring triterpenoid, which can be indicated in the rhizome of medicinal and dietary plants from Alisma species. Previous studies have demonstrated that AB23A could inhibit intestinal permeability by regulating tight junction (TJ)-related proteins. Even so, the AB23A protective mechanism against intestinal barrier dysfunction remains poorly understood. This investigation seeks to evaluate the AB23A protective effects on intestinal barrier dysfunction and determine the mechanisms for restoring intestinal barrier dysfunction in LPS-stimulated Caco-2 monolayers. According to our findings, AB23A attenuated the inflammation by reducing pro-inflammatory cytokines production like IL-6, TNF-α, IL-1β, and prevented the paracellular permeability by inhibiting the disruption of TJ in LPS-induced Caco-2 monolayers after treated with LPS. AB23A also inhibited LPS-induced TLR4, NOX1 overexpression and subsequent ROS generation in Caco-2 monolayers. Transfected with NOX1-specific shRNA diminished the up-regulating AB23A effect on ZO-1 and occludin expression. Moreover, transfected with shRNA of TLR4 not only enhanced ZO-1 and occludin expression but attenuated NOX1 expression and ROS generation. Therefore, AB23A ameliorates LPS-induced intestinal barrier dysfunction by inhibiting TLR4-NOX1/ROS signaling pathway in Caco-2 monolayers, suggesting that AB23A may have positive impact on maintaining the intestinal barrier’s integrity.

## Introduction

The increase in gut permeability is the most prominent feature resulting from intestinal barrier dysfunction, which may facilitate the passage of foreign antigens and pathogens across the epithelial barrier (Arrieta et al., 2006). Tight junctions (TJs) are vital for constructing a barrier between intestinal epithelial cells at the epithelium’s apical regions and serve as paracellular gates that restrict diffusion depending on size and charge, maintaining organ and tissue homeostasis (Zihni et al., 2016). Numerous TJ proteins have thus been found, such as cytoplasmic proteins zonula occludens-1 (ZO-1), the transmembrane proteins occluding and claudins (Clark et al., 2006). The gut TJ barrier disruption leads to a “leaky” TJ barrier, resulting in a rise in gut permeability, and permitting paracellular permeation of luminal antigens that enhance intestinal inflammation, which is associated with various diseases comprising irritable bowel syndrome, diabetes, coeliac disease, non-alcoholic fatty liver disease, Crohn’s disease and so on (Barbara et al., 2021; Portincasa et al., 2021; Prospero et al., 2021; Riedel et al., 2021; Xu et al., 2021).

In Gram-negative bacteria, lipopolysaccharide (LPS) is not only a component of the cell wall but one of the leading causes of the pathogenesis of septic shock and endotoxemia (Wang et al., 2019). Toll-like receptors (TLRs) play a vital role in inflammation provoked by diverse stimuli as the primary receptors of innate immunity, resulting in activating the inflammatory pathway, such as TLR2, TLR4, and so on (Wang et al., 2017). Among them, TLR4 was the initial TLR discovered in humans, which is responsible for recognizing bacterial LPS, initiating endotoxin-mediated inflammation (Miyake, 2004). Numerous studies have found that LPS-induced low-grade chronic systemic inflammatory will lead to a significant alteration in the function and key TJ proteins expression (Tao et al., 2017; Feng et al., 2022). Inhibiting LPS-mediated activation of TLR4 and subsequent inflammation pathway might be a useful way to against the impairment of intestinal barrier.

Excessive reactive oxygen species (ROS) production and cell redox imbalance have been shown to have a key role in the pathophysiology of the inflammatory response (Veith et al., 2019). NADPH oxidase (NOX) was firstly recognized as a main source of ROS in immune cells; and recently has been revealed to have a vital role in inflammation-related-cellular signalling, including NOX1, NOX2, NOX3, NOX4, NOX5, and DOUX1 and DOUX2 (Vermot et al., 2021). Among all these homologs, NOX1 is abundantly expressed in the gastrointestinal tract and has been implicated in inflammatory responses and local innate immune (Geiszt et al., 2003a; Juhasz et al., 2017). Recent studies demonstrated a pivotal role of NOX1-derived ROS in gut intestinal epithelial homeostasis and barrier functions regulation, indicating NOX1, a reactive oxygen species (ROS)-producing oxidase might be important for endotoxin-induced intestinal barrier dysfunction (Geiszt et al., 2003b; Yasuda et al., 2012; Yokota et al., 2017).

Rhizoma alisamatis (Ze xie in Chinese, Takusha in Japanese, and Taeksa in Korean, AR) is one of a widely used medicinal and dietary plants in some East Asian countries (Jang and Lee, 2021). Alisol B 23-acetate (AB23A) was isolated from the rhizome of Rhizoma alisamatis as a natural triterpenoid and has been proven to show outstanding bioactivity, comprising anti-hyperlipidemia, hepatoprotective, and anti-inflammatory (Meng et al., 2015; Wang et al., 2019). Moreover, A novel study indicated that AB23A enhances intestinal permeability and microecological disorders in mice with colitis-associated cancer (CAC) (Zhu et al., 2021). Our previous study also has confirmed that AB23A could inhibit high fat diet-induced down-regulation of TJ-related proteins in NAFLD mice (Xia et al., 2021). Even so, AB23A mechanism on intestinal permeability is poorly known. Hence, assessing the protective effects and basic AB23A mechanism on intestinal barrier function in Caco-2 monolayers was the objective of this investigation.

## Materials and Methods

### Reagent

Chengdu Must Biotechnology Co., Ltd. (Chengdu, China) and Beyotime Biotechnology (Jiangsu, China) provided AB23A (purity > 98%) (Supplementary Figure S1) and LPS from *E. coli* (0111: B4), respectively. AB23A stock solutions were made in 0.1% DMSO, and working solutions were made in a culture medium followed by filter sterilization (0.2 mm) just prior to utilization. Gibco Life Technologies provided Dulbecco’s Modified Eagle’s Medium (DMEM) culture medium and fetal bovine serum (FBS). All biochemical indicator kits and other chemicals used in the study were commercially available unless otherwise mentioned.

### Cell Culture

Human Caco-2 cells were graciously donated by the Chinese Academy of Sciences’ Cell Bank/Stem Cell Bank (Shanghai, China) and cultured in DMEM supplemented with 10% FBS, 100 units/mL penicillin, 100 mg/ml streptomycin. The cells were cultured at 37°C in a humidified incubator containing 5% CO_2_ and routinely trypsinized. Every 2–3 days, the culture media was replaced.

### Cell Viability Assessment

The Caco-2 cells were seeded at a density of 1 × 10^4^ cells/well per 100 μl in 96-well plates and underwent incubation overnight, and then underwent treatment with various concentrations (0.1, 1, 10, and 100 μg/ml) of LPS or AB23A (0, 0.625, 1.25, 2.5, 5, 10, 20, and 40 μM) in a humidified incubator with 5% CO_2_ at 37°C for 12 h. The culture media was collected following treatment, and 100 μl of 1/10 (vol/vol) CCK-8 reagent in medium was added per each well. After 2 h of incubation at 37°C, the absorbance was measured at 450 nm utilizing a Synergy H1 multifunctional microplate reader (Biotek, United States). The cell viability was estimated by dividing the sample’s optical density by the control group’s optical density.

### Intestinal Barrier Permeability Assay

The establishment of intestinal barrier *in vitro* and the permeability assay were performed in accordance with the past investigation (Hubatsch et al., 2007). Briefly, prior to the trials, on a permeable polycarbonate membrane 24-Transwell supporting system with 0.4 um holes, cells were plated at a density of 5 × 10^4^/cm^2^. (Corning, United States). To confirm the integrity of Caco-2 monolayers cultured under static conditions *in vitro*. Transepithelial electrical resistance (TEER) was measured every 2 days starting from day 8 with an ohm/volt meter 2 (EVOM2; World Precision Instruments, United States) in compliance with the manufacturer’s instructions. In accordance with the previous study, when the cells attained confluence (TEER > 300 Ω cm^2^) (Hubatsch et al., 2007), various concentrations (2.5, 5 and 10 μM) of AB23A were added to the cells with or without 10 μg/ml LPS for 12 h. Following treatment, apical to basolateral flux of 10 kDa fluorescein isothiocyanate-labeled dextran (FITC-dextran) was utilized for assessing barrier function. FITC-dextran (100 μl, 1 mg/ml) was introduced to the apical chamber, and then the basolateral medium was collected after 2 h. The fluorescence in the basolateral medium was measured utilizing a Synergy H1 multifunctional microplate reader (Biotek, United States) at 427 nm excitation and 536 nm emission. The apparent permeability coefficient (Papp) was calculated according to the equation (Hubatsch et al., 2007):

$$\mathrm{Papp}\left(\mathrm{cm}/s\right)=\frac{\mathrm{dQ}/\mathrm{dt}}{AC_{0}}\;$$

A = filter surface (0.33 cm^2^), C_0_ = initial concentration of FITC-dextran in the apical chamber (1 mg/ml), dQ/dt = amount of FITC-dextran (μg/s).

### Immunofluorescence Assay

Cells were cultured on coverslips in 6-well plates at a density of 3 × 10^5^ per well. Monolayer of cells forms after about 9–12 days, various concentrations of AB23A (2.5, 5, and 10 μM) were added with or without 10 μg/ml LPS for 12 h. Following three washes with PBS. The cells were kept in 4% paraformaldehyde for 10 min before being permeabilized in 0.1% Triton X-100 in PBS for 15 min (ZO-1) or 10 min (occludin) at room temperature. Then cells were blocked for 1 h at room temperature with 2% BSA/PBS, and underwent incubation with an anti-ZO-1 (1:50, Invitrogen, United States) or occludin (1:125, Invitrogen, United States) antibodies at 4°C overnight. The cells were rinsed with PBS (3 × 10 min) and then underwent incubation with FITC-conjugated goat anti-rabbit IgG secondary antibody (1:200, Affinity, United States) at room temperature for 45 min. After further washing with PBS (3 × 10 min), nuclei were counterstained with a 1:10,000 dilution of 4′, 6-diamidino-2-phenylindole (DAPI, Beyotime Biotechnology, Shanghai, China) at room temperature for 2 min. Excess dye was removed by rinsing three times with PBS (3 × 10 min). Anti-fluorescence quenching solution (Beyotime Biotechnology, Shanghai, China) was dropped on the slide. Coverslips were placed onto slides and then detected with confocal laser scanning microscope (LSM 880 with AiryScan, Zeiss, Germany).

### ROS Generation Analysis

In compliance with the manufacturers’ instructions, the ROS generation was evaluated utilizing the cellular ROS assay kit (Abcam, Cambridge, United Kingdom). Cells underwent incubation with 10 μM 2′–7′-dichlorofluorescin diacetate (DCFH-DA) at 37°C in the dark for 30 min. By measuring intracellular ROS, the fluorescence density of 2′–7′-dichlorfluorescin (DCF) was detected utilizing Synergy H1 multifunctional microplate reader (Biotek, United States) or fluorescence microscope (Leica DMI8, Germany).

### Enzyme-Linked Immunosorbent Assays

Utilizing an Enzyme-Linked Immunosorbent Assays (ELISA) kit, the pro-inflammatory cytokines production comprising IL-6, TNF-, and IL-1 from Caco-2 cells was detected (Mutisciences, Hangzhou, China). Briefly, Caco-2 cells underwent treatment with varying concentrations of AB23A (2.5, 5, and 10 M) with or without 10 μg/ml LPS for 12 h. In compliance with the manufacturer’s instructions, IL-6, TNF-α, and IL-1β concentrations were evaluated by collecting cell supernatants. Absorbance was read utilizing Synergy H1 multifunctional microplate reader at 450 nm (Biotek, United States).

### Transfection With Short Hairpin RNA

GeneChem provided the plasmid utilized in TLR4 and NOX1-specific Short Hairpin RNA (shRNA)-mediated knockdown experiments (Shanghai, China). The negative control was constructed utilizing an empty plasmid (sh-Ctrl). Cells were seeded at the density of 5 × 10^5^ per well in 6-well plates. In compliance with the manufacturer’s instructions, plasmids were transiently transfected into cells by utilizing a Lipo3000 kit (Invitrogen, Shanghai, China). Following gene transfection, the cells were subsequently cultured for 48 h before being treated with or without LPS and AB23A as mentioned above.

### RNA Isolation and qPCR

In compliance with the manufacturer’s instructions, the SteadyPure Universal RNA Extraction Kit II was utilized for isolating total RNA (Accurate biotechnology Co., Ltd., Shenzhen, China). Nanodrop ND-8000 was utilized for assessing RNA concentration and purity (Thermo Fisher Scientific, Waltham, MA, United States). Then, the RNA was reverse transcripted to cDNA utilizing the Evo M-MLV RT Premix (Accurate biotechnology Co., Ltd., Shenzhen, China). Transcript levels of TLR4, NOX1, and β-actin were quantified utilizing the SYBR Green Premix Pro Taq HS qPCR Kit (Accurate biotechnology Co., Ltd., Shenzhen, China) on a CFX96 Real-Time System (Bio-Rad, United States). Table 1 demonstrates the primers utilized for different genes amplification. The quantity of mRNA was normalized with β-actin as an internal standard.

**TABLE 1:** Primer sequences of NOX1, TLR4 and β-actin in this study.

Gene	Sequences (5′ → 3′)
NOX1	F: TCT​TAA​AGG​CTC​ACA​GAC​CCT​G
	R: CAG​CCC​TAA​CCA​AAC​AAC​CAG​AA
TLR4	F: GGA​GAG​AAA​GAC​ACC​GAG​AAT​G
	R: CCC​AAG​GCA​CAC​AGT​TGA​TA
β-actin	F: TGG​CAC​CCA​GCA​CAA​TGA​A
	R: CTA​AGT​CAT​AGT​CCG​CCT​AGA​AGC​A

### Western Blot Analysis

Each well was emptied of its cell culture medium and rinsed twice with PBS. In compliance with the manufacturer’s instructions, RIPA lysis buffer was utilized for extracting total proteins (Beyotime, Shanghai, China). Utilizing BCA protein assays, all samples were analyzed (Thermo, United States). Protein supernatant was combined with sodium dodecyl sulfate-polyacrylamide gel electrophoresis (SDS-PAGE) loading buffer and immersed for 5 min in a boiling water bath. Following standard protocols, equal quantities of proteins were separated with 8%, 10%, or 12% SDS-PAGE gels and afterward transferred to a PVDF membrane (Millipore, United States). The PVDF membrane was blocked with 5% non-fat milk for 2 h, and underwent incubation overnight at 4°C with primary antibodies, including anti-human polyclonal antibodies that recognize TLR4 (1:1,000, Invitrogen, United States), NOX-1 (1:1,000, Proteintech Group, Wuhan, China), ZO-1 (1:500, Invitrogen, United States), occludin (1:500, Invitrogen, United States) and β-actin (1:2,000, Beyotime Biotechnology, Jiangsu, China), At room temperature, to mix the primary antibodies for 1–2 h, the secondary goat anti-rabbit horseradish peroxidase (HRP)-IgG antibody (1:2,000, Affinity, United States) was added. ECL (Beyotime Biotechnology, Shanghai, China) was utilized for visualizing proteins under the iBright intelligent imaging system FL1000 (Invitrogen, United States).

### Statistical Analysis

One-way analysis of variance (ANOVA) was followed by Tukey’s multiple comparisons test or Newman-Keuls multiple comparisons test and Student’s *t* test with GraphPad Prism software to analyze group differences (version 8.3.0). The data were reported as mean ± standard deviation (SD). *p* < 0.05 indicated statistically significant differences.

## Results

### LPS and AB23A Effects on Caco-2 Cell Viability

**FIGURE 1:**
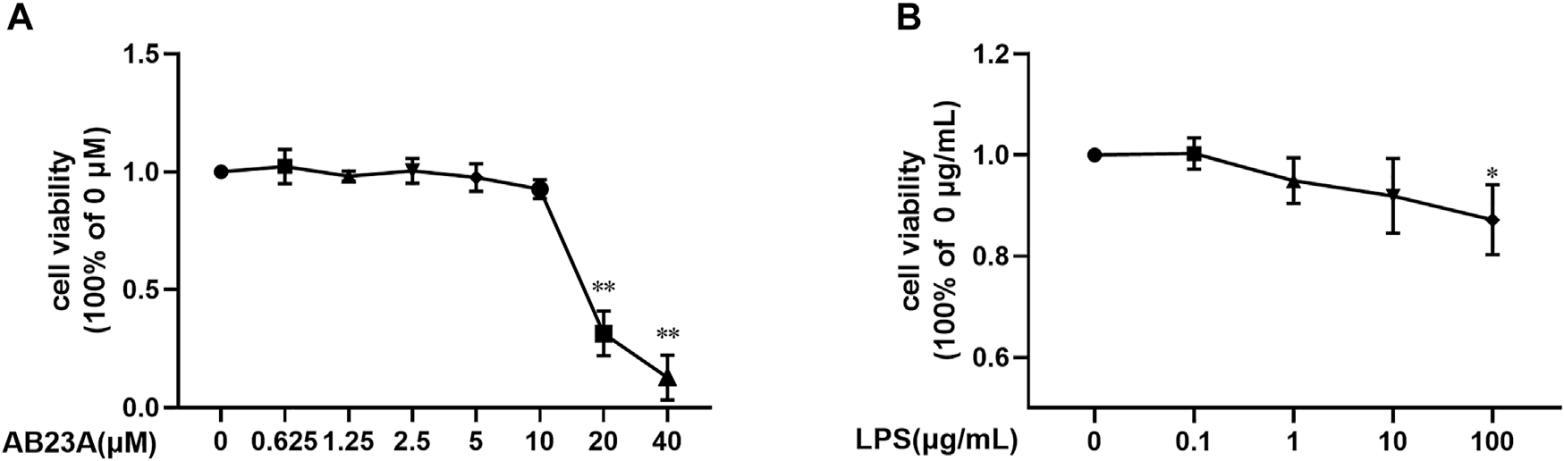
LPS and AB23A effects on the cell viability of Caco-2 cells. Caco-2 cells underwent treatment with various AB23A or LPS concentrations for 12 h. **(A)** Effect of different concentrations of LPS in Caco-2 cell viability. **(B)** Effect of various AB23A concentrations on Caco-2 cell viability. **p < 0.01 vs. AB23A (0 μM) or LPS (0 μg/ml).

CCK-8 assay was conducted to evaluate cytotoxicity. As shown in Figure 1, the relative viabilities of the cell treated with 100 μg/ml of LPS were significantly lower compared to the group of LPS (0 μg/ml). For AB23A treatment, 20, 40 μM of AB23A caused a marked cytotoxicity on Caco-2 cells. Therefore, we selected 10 μg/ml of LPS and 2.5, 5, and 10 μM of AB23A in further assays (Figures 1A,B).

### AB23A Inhibits LPS-Induced Pro-Inflammatory Cytokines Expression in Caco-2 Cells

**FIGURE 2:**
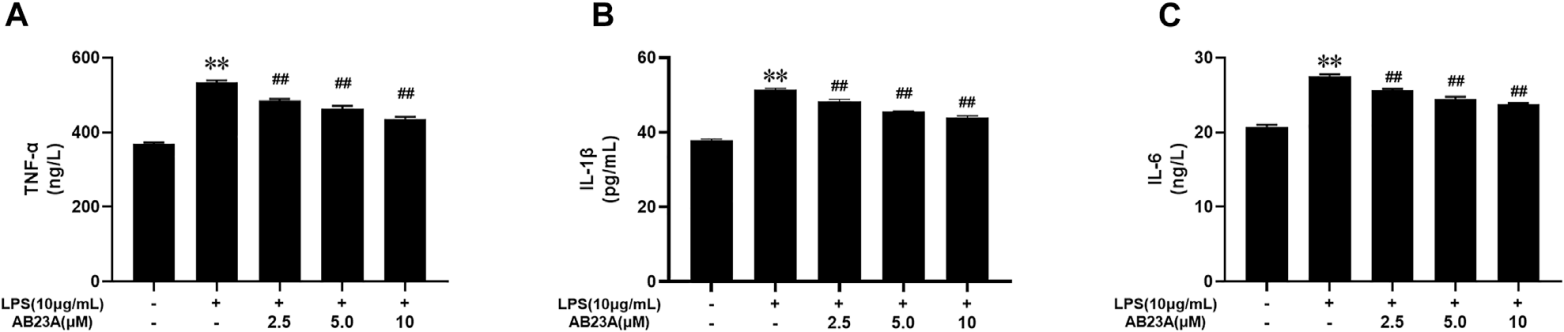
AB23A effects on the TNF-α, IL-6 and IL-1β levels induced by LPS in Caco-2 cells. Caco-2 cells underwent treatment with various AB23A concentrations (2.5, 5 and 10 μM) with or without 10 μg/ml LPS for 12 h. **(A)** TNF-α level. **(B)** IL-6 level. **(C)** IL-1β level. **p < 0.01 vs. control; ^##^ *p* < 0.01 vs. LPS.

By ELISA, TNF-α, IL-6, and IL-1β concentrations in the cell supernatants were detected. LPS alone enhanced TNF-α, IL-6, and IL-1β expression levels in Caco-2 cells significantly. However, AB23A dose dependently reduced the LPS-induced elevation in TNF-α, IL-6, and IL-1β levels in a (Figures 2A–C). Similar with result of AB23A on AOM/DSS-Induced CAC Mice (Zhu et al., 2021), these findings revealed that AB23A could inhibit LPS-induced pro-inflammatory cytokines expression in Caco-2 cells.

### AB23A Attenuates LPS-Induced ROS Generation in Caco-2 Cells

**FIGURE 3:**
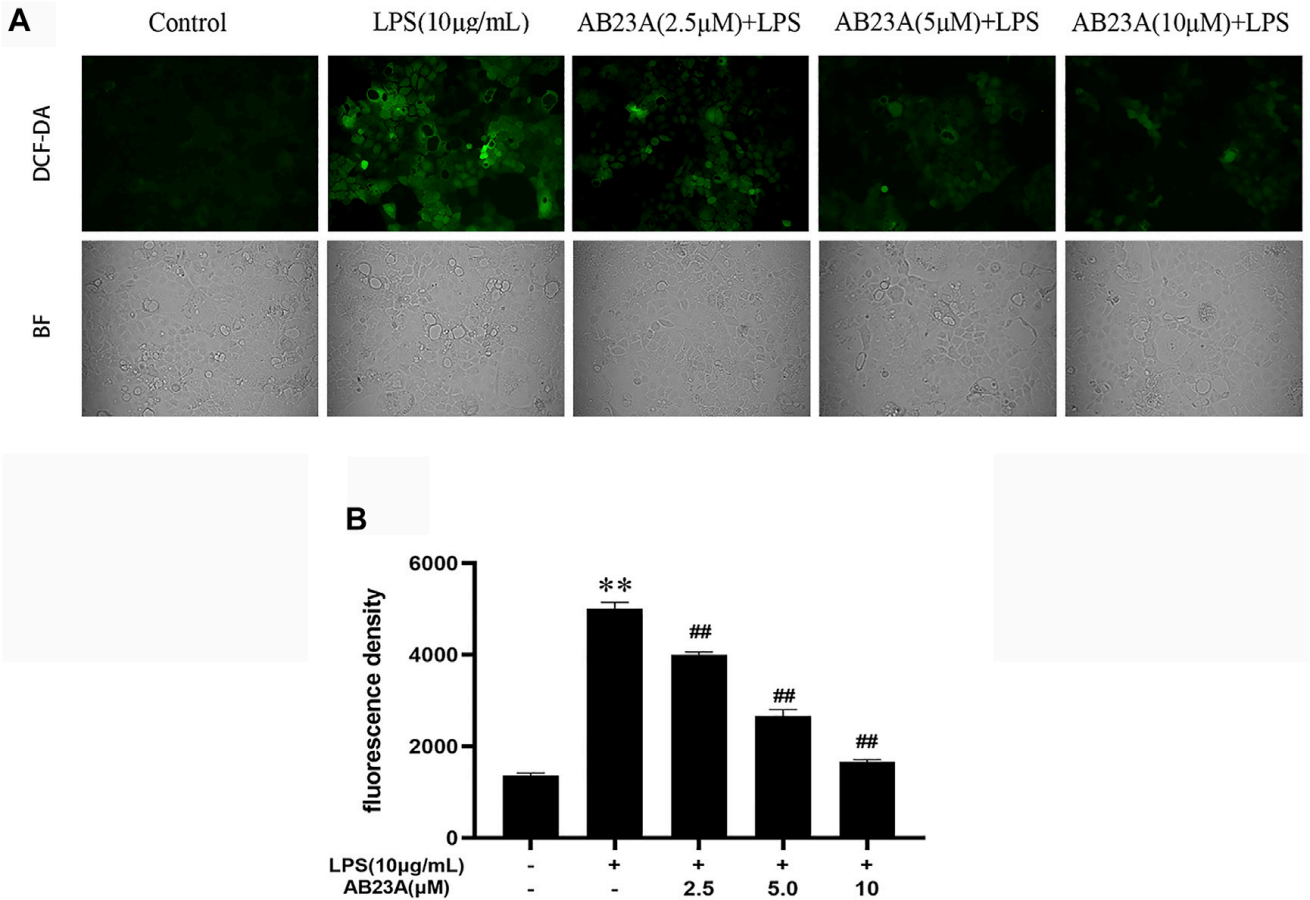
AB23A effect on the ROS generation induced by LPS. Caco-2 cells underwent treatment with AB23A concentrations (2.5, 5 and 10 μM) with or without 10 μg/ml LPS for 12 h. **(A)** Utilizing a fluorescent microscope, intracellular ROS was noticed, and the corresponding cell morphology was captured utilizing a bright field (BF) (magnification, ×10). **(B)** ROS were detected under a multifunctional microplate reader. **p < 0.01 vs. control; ^##^ *p* < 0.01 vs. LPS (10 μg/ml).

DCFH-DA staining with fluorescence microscopy was utilized for measuring ROS production. The intensity of green fluorescence indicated the level of ROS generation in Caco-2 cells. The LPS-treated cells presented a stronger green fluorescence intensity at the cell edges and inside of the cells than that observed in control group. While, AB23A attenuated the intensity of green fluorescence compared with LPS treatment group (Figure 3A). As evident from the Figure 3B, the attenuating effects of AB23A (2.5, 5, and 10 μM) on ROS generation (mean fluorescent intensity: 4,014.00 ± 59.27, 2,704.00 ± 120.76, and 1,677.00 ± 32.54, respectively) were observed compared to that noted in the LPS-induced cells (mean fluorescent intensity: 5,048.50 ± 123.70). These findings revealed that LPS-induced intracellular ROS generation is significantly attenuated by AB23A in a concentration-dependent manner.

### AB23A Inhibits LPS-Induced Intestinal Barrier Permeability in Caco-2 Cells

**FIGURE 4:**
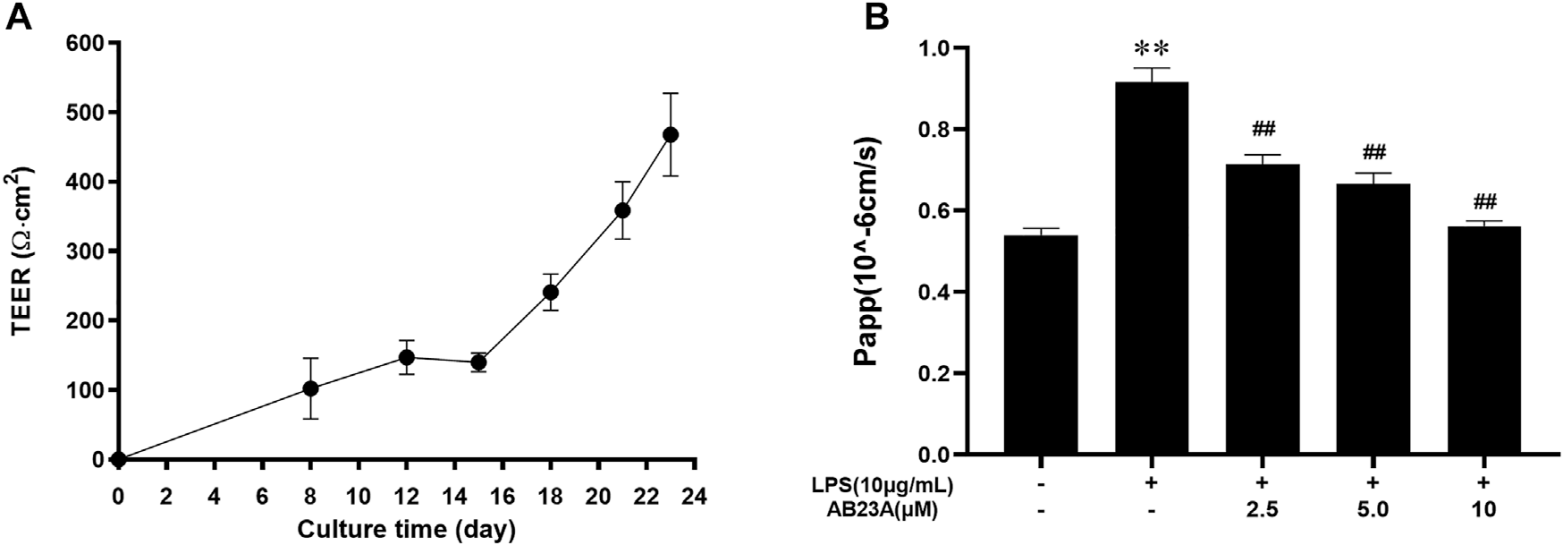
AB23A effects on intestinal barrier permeability induced by LPS. **(A)** At various time points, the transepithelial electrical resistance (TEER) was assessed. **(B)** The apparent permeability coefficient (Papp) test was utilized for assessing intestinal permeability *in vitro*. **p < 0.01 vs. control; ^##^ *p* < 0.01 vs. LPS (10 μg/ml).

For examining AB23A effect on intestinal barrier function, a vitro model was utilized where Caco-2 epithelial cell monolayers underwent treatment with LPS (10 μg/ml). As shown in Figure 4A, TEER increased with the increase of culture time, and the mean TEER reached 467.8 Ω cm^2^ on day 23, revealing an *in vitro* model of the intestinal barrier for Caco-2 cells was successfully established. LPS (10 μg/ml) elevated apical to basolateral flux of FITC-dextran (Hubatsch et al., 2007). Moreover, AB23A effectively reduced LPS-induced the apical to basolateral flux of FITC-dextran in Caco-2 epithelial cell monolayers (Figure 4B). These results suggested that AB23A enhances barrier integrity in Caco-2 monolayers.

### AB23A Alleviates LPS-Induced Abnormal Structural Changes and Distribution of TJ in Caco-2 Cells

**FIGURE 5:**
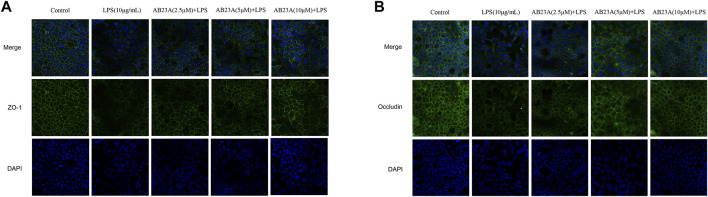
AB23A effects on LPS-induced TJ structure and distribution in Caco-2 cells. Cells were cultured in the medium for several days before being treated with or without LPS (10 μg/ml) and various concentrations of AB23A (2.5, 5 and 10 μM) for 12 h. Immunofluorescence staining of **(A)** ZO-1 and **(B)** occludin. DAPI staining of the nuclei was utilized as a control image. Confocal laser scanning microscope was utilized to observe ZO-1and occludin protein localization in Caco-2 cells with or without LPS and AB23A (magnification, ×20), respectively.

For evaluating AB23A effect on the intercellular distribution and occludin and ZO-1 expression, an immunofluorescence assay of Caco-2 cells with a specific antibody against occludin and ZO-1 was undertaken. In control group, results revealed that TJ proteins showed a normal, organized structure and well-characterized localization around the cell boundaries, whereas the LPS-treated cells had fainter and abnormal structural staining at the same location. AB23A (2.5, 5, and 10 μM)-treated cells showed a stronger staining, compared with that of the LPS treatment alone (Figures 5A,B). These results indicated that AB23A might enhance the barrier integrity by restore TJ structure and distribution in Caco-2 monolayers.

### AB23A Inhibits LPS-Induced TLR4 and NOX1 Expression in Caco-2 Cells

**FIGURE 6:**
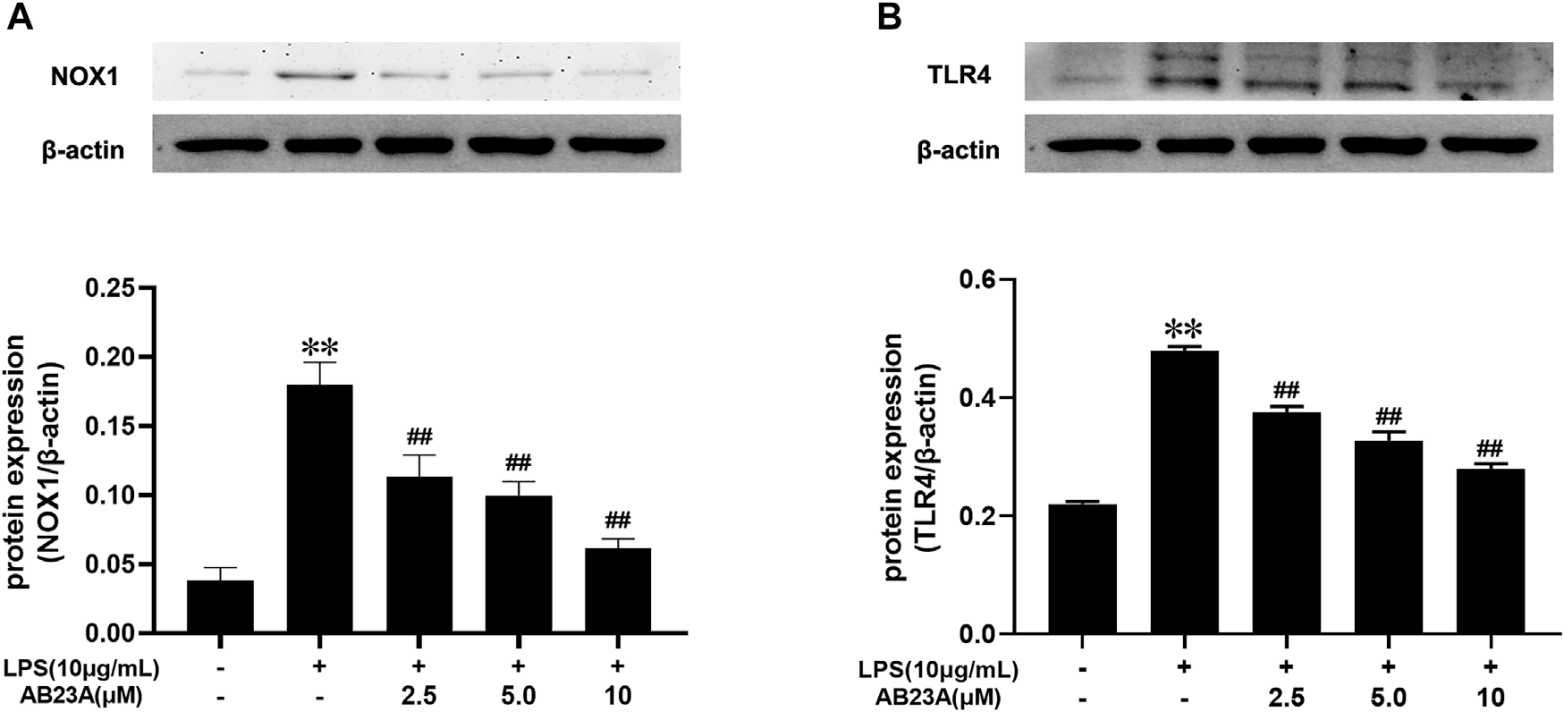
AB23A effects on the TLR4 and NOX1 proteins expression induced by LPS. **(A)** The TLR4 protein expression level. **(B)** The NOX1 protein expression level. All gel images are from the same sample. β-actin was utilized repeatedly as a control image. **p < 0.01 vs. control; ^##^ *p* < 0.01 vs. LPS (10 μg/ml).

LPS alone dramatically elevated the TLR4 and NOX1 protein expression com-pared to that observed in the control. While, AB23A attenuated TLR4 and NOX1 proteins expression stimulated by LPS in a dose-dependently manner (Figures 6A,B). Consequently, AB23A downregulated the TLR4 and NOX1 proteins expression induced by LPS.

### AB23A Attenuates LPS-Induced ROS Generation by Inhibiting NOX1 Expression in Caco-2 Cells

**FIGURE 7:**
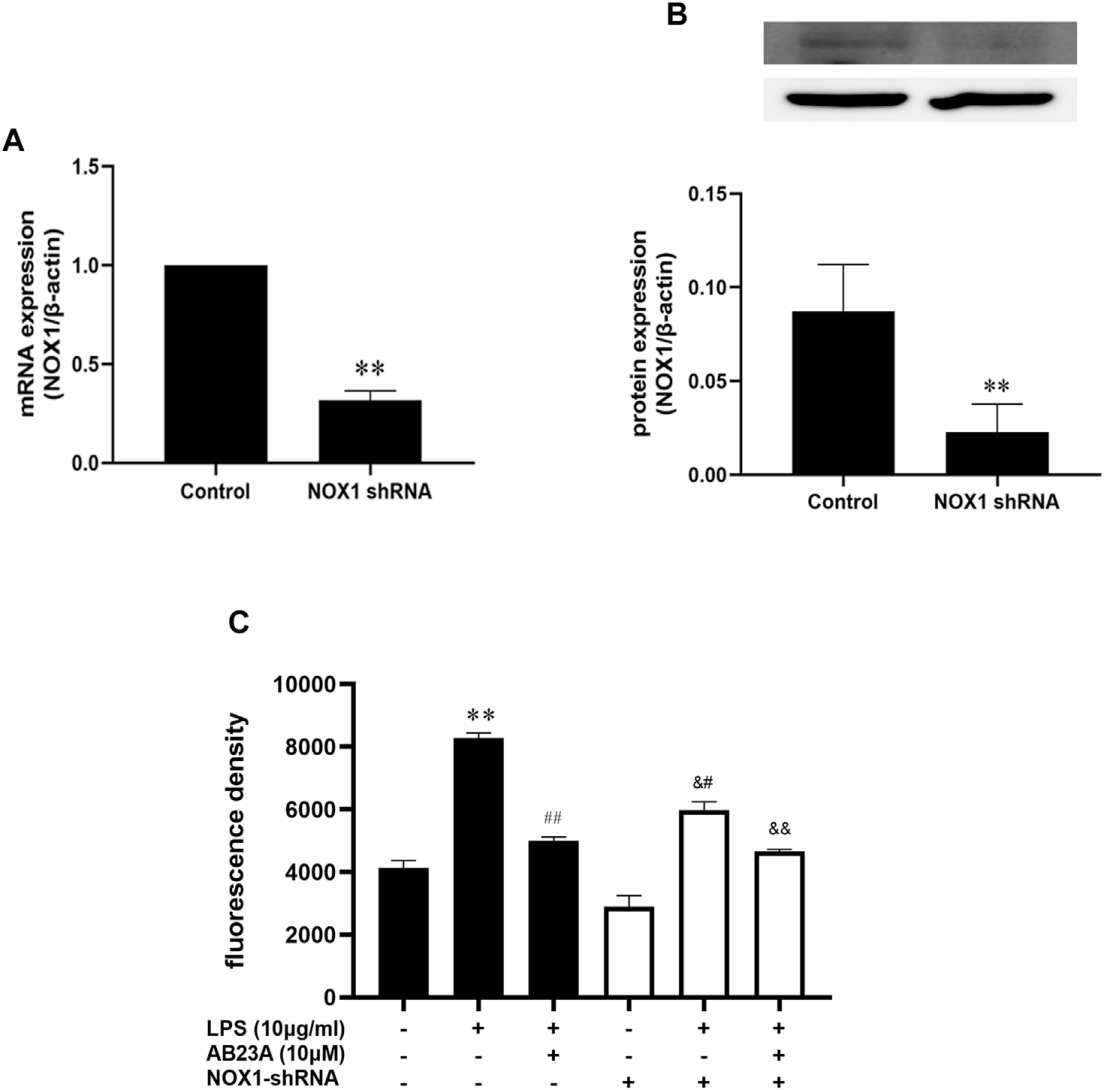
AB23A effects on the ROS generation induced by LPS in TLR4-shRNA transfected cells. **(A)** The NOX1 mRNA expression level. **(B)** The NOX1 protein expression level. **(C)** AB23A effects on the ROS generation induced by LPS in NOX1 knockdown cells. **p < 0.01 vs. control; ^##^ *p* < 0.01 vs. LPS (10 μg/ml); ^&#^ *p* < 0.01 vs. NOX1-shRNA; ^&&^ *p* < 0.01 vs. NOX1-shRNA + LPS (10 μg/ml).

To further evaluate whether the reduction of ROS by AB23A was involved in the suppression of the NOX1 expression, we transfected shRNA of NOX1 into Caco-2 cells and measured the ROS production. The expressions of NOX1 on mRNA and protein level were significantly decreased when NOX1 was knocked down by shRNA against NOX1 (Figures 7A,B). Additionally, AB23A significantly attenuated the ROS generation by 1.66-fold decrease compared to the observed with LPS treatment alone in normal Caco-2 cells (ROS mean fluorescence intensity induced by LPS = 8,287.0 ± 160.87; ROS mean fluorescence intensity induced by LPS + AB23A = 5,003.3 ± 117.32). However, following NOX1-shRNA transfection, the attenuating effect of AB23A on LPS-induced ROS generation was diminished (ROS mean fluorescence intensity induced by LPS = 5,985.3 ± 258.75; the mean fluorescence intensity of ROS induced by LPS + AB23A = 4,664.8 ± 67.43; fold decrease = 1.28). These results suggested that AB23A attenuates LPS-induced ROS generation by inhibiting NOX1 expression.

### AB23A Attenuates LPS-Induced Intestinal Barrier Permeability by Inhibiting NOX1/ROS Expression in Caco-2 Cells

**FIGURE 8:**
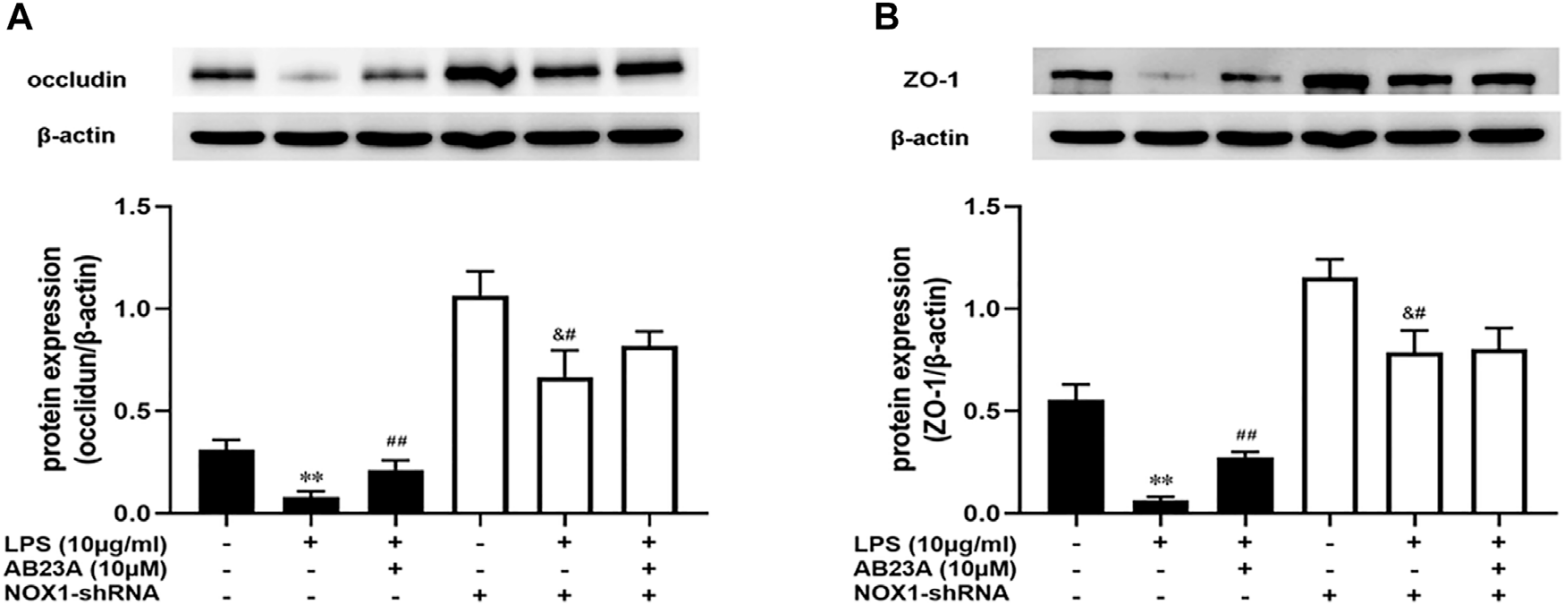
AB23A effects on the intestinal barrier permeability induced by LPS in association with the inhibition of NOX1/ROS. Following transfection with NOX1 shRNA, the cells underwent treatment with or without LPS and AB23A for 12 h. **(A)** The occludin and ZO-1 protein expression levels. **(B)** ZO-1 protein expression level. All gel images are from the same sample. β-actin was utilized repeatedly as a control image. **p < 0.01 vs. control; ^##^ *p* < 0.01 vs. LPS (10 μg/ml); ^&#^ *p* < 0.01 vs. NOX1-shRNA.

To determine if AB23A attenuates LPS-induced intestinal barrier permeability by inhibiting NOX1/ROS expression, we selectively silenced NOX1. As demonstrated in Figure 8, In comparison with the control group, LPS significantly diminished the occludin and ZO-1 proteins expression levels and AB23A treatment elevated the occludin and ZO-1 proteins expressions levels induced by LPS. While, after transfection of Caco-2 cells with NOX1 shRNA, AB23A treatment has no significant difference compared to that were treated with LPS alone on LPS-induced occludin and ZO-1 proteins expression, demonstrating the increased effect of AB23A was abolished on LPS-induced occludin and ZO-1 proteins expression. Therefore, we speculated that AB23A might attenuate LPS-induced intestinal barrier permeability by inhibiting NOX1/ROS expression in Caco-2 cells.

### TLR4-NOX1/ROS Axis Might Play a Key Role in LPS-Induced Intestinal Barrier Permeability in Caco-2 Cells

**FIGURE 9:**
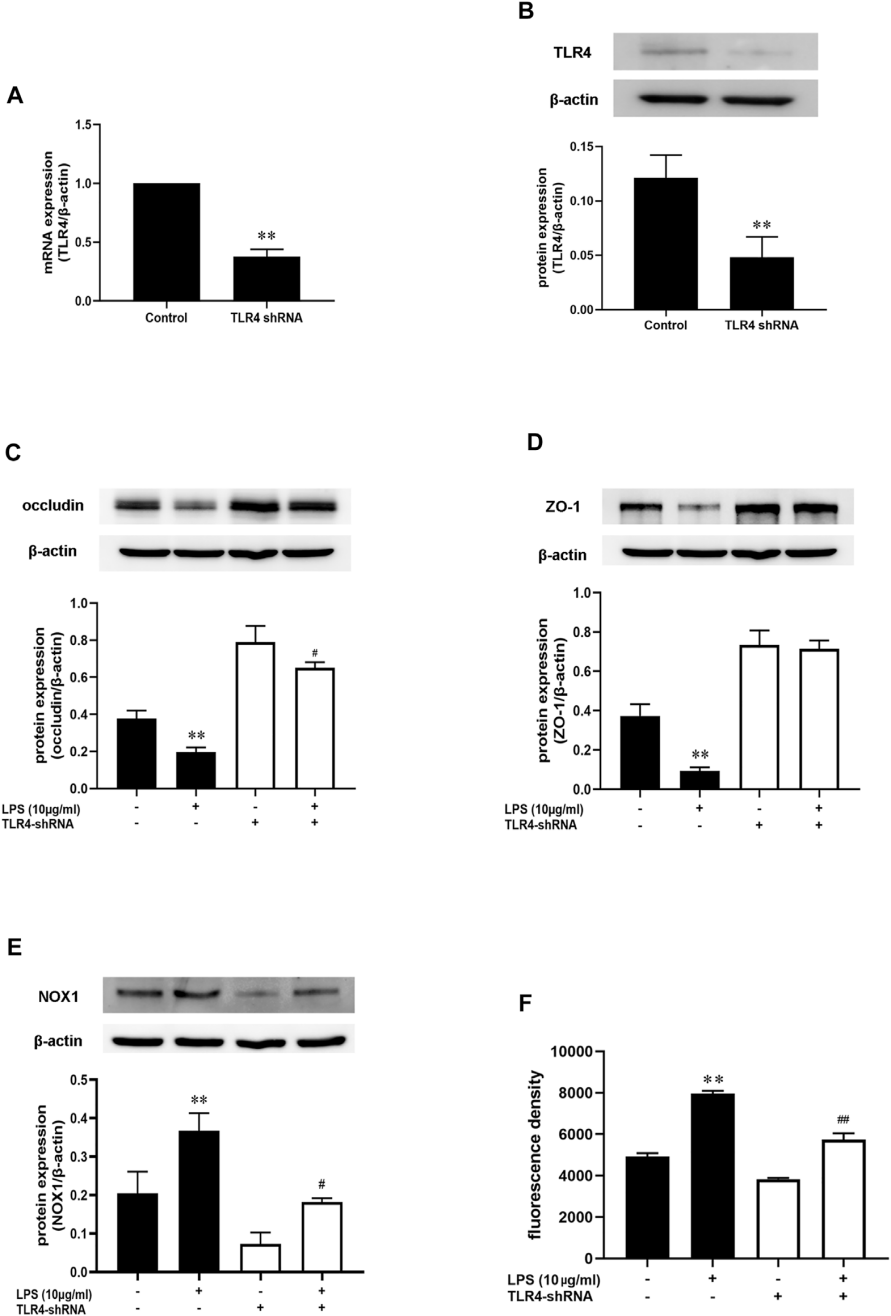
AB23A effects on the LPS-induced intestinal barrier permeability by inhibiting the TLR4-NOX1/ROS pathway. Following transfection with TLR4 shRNA, the cells were subsequently cultured for 48 h before being treated with LPS for 12 h. **(A,B)** The mRNA and protein expression level of TLR4. **(C,D)** The occludin and ZO-1 protein expression level. **(E)** The NOX1 protein expression level after transfected with TLR4-shRNA with or without LPS stimulation. **(F)** The ROS production following transfection with TLR4-shRNA with or without LPS stimulation. All gel images are from the same sample. β-actin was utilized repeatedly as a control image. **p < 0.01 vs. control; ^#^ *p* < 0.05 vs. TLR4-shRNA + LPS (10 μg/ml); ^##^ *p* < 0.01 vs. TLR4-shRNA + LPS (10 μg/ml).

Above data demonstrated that AB23A effect on intestinal barrier permeability was associated with inhibiting NOX1/ROS expression. AB23A also showed a significant inhibition of both TLR4 and NOX1 during LPS induction. However, the inter-action of TLR4 with NOX1/ROS on intestinal barrier permeability remains unknown. As shown in Figures 9A,B, TLR4 expressions on mRNA and protein level were significantly decreased after treatment with TLR4-shRNA. In comparison with that in the control group, LPS alone diminished the occludin and ZO-1 proteins expression levels while transfection with TLR4-shRNA inhibited the decrease of occludin and ZO-1 protein expressions induced by LPS, suggesting that TLR4 might have a vital role in LPS-induced intestinal barrier permeability (Figures 9C,D). More importantly, the TLR4-shRNA down-regulated NOX1 protein expression and ROS generation with or without LPS stimulation (Figures 9E,F). Therefore, we hypothesized that TLR4-NOX1/ROS might play a key role in LPS-induced intestinal barrier permeability in Caco-2 cells.

## Discussion

Fatty liver diseases comprising steatohepatitis (NASH) and non-alcoholic fatty liver disease (NAFLD) are related to elevated intestinal barrier permeability and translocation of bacteria or bacterial products into the blood circulation, according to accumulating studies (Mouries et al., 2019). Past investigations have demonstrated that AB23A maintains the intestinal barrier integrity and reduces the endotoxin level in HFD-induced NAFLD or colitis-associated cancer (CAC) mice (Xia et al., 2021; Zhu et al., 2021). Even so, AB23A protective mechanisms on the function of the intestinal barrier remain poorly understood.

Tight junctions (TJs) are vital for establishing a barrier between various compartments of the body, and their major physiological purpose is to serve as paracellular gates that restrict diffusion on the basis of size and charge (Zihni et al., 2016). The disruption of intestinal epithelial TJ formation by pathogens is one of the most critical factors in determining gut permeability, such as pathogenic bacteria, LPS, inflammatory mediators (Ling et al., 2016). Disruption of the intestinal barrier is present in a wide range of gut-associated diseases. As the first TJ-associated protein to be discovered, ZO-1 is widely regarded as an effective marker for identifying intact cell-to-cell connections and evaluating TJ integrity (Montalto et al., 2004). Occludin is an integral membrane protein particularly linked with tight junctions, which is directly encompassed in cell-cell adhesion and colocalize with ZO-1, maintaining intestinal barrier integrity (Van Itallie and Anderson, 1997; Arrieta et al., 2006; Zihni et al., 2016). More importantly, signaling at tight junctions appears to have a critical role in the cellular stress response (Nusrat et al., 2000; Barrios-Rodiles et al., 2005; Lockwood et al., 2008). In our *in vitro* investigation, we reveal that LPS stimulation not only decreases occludin and ZO-1expression, but also affects occludin and ZO-1 proteins’ location in Caco-2 monolayers, resulting in enhanced intestinal permeability. AB23A displays a protective effect on intestinal permeability by upregulating and restoring occludin and ZO-1 expression and distribution. These findings are consistent with our previous results in NAFLD mice showing that AB23A maintains intestinal barrier integrity by inhibiting HFD-induced downregulation of TJ expressions, including ZO-1 and occludin (Xia et al., 2021).

By activating redox-sensitive protein kinases and transcription factors, ROS has been identified as essential signaling molecules that regulate the transcription of several genes (Cakir and Ballinger, 2005; Yasuda et al., 2012). NOX has been implicated to be the major source of ROS generation in the pathogenesis of gut-associated diseases (Yokota et al., 2017). More importantly, NOX1 is more abundantly expressed than other isoforms throughout the gastrointestinal tract, and has been implied to play a role in local innate immunological and inflammatory responses (Rokutan et al., 2006; Kamizato et al., 2009). Thus, we speculated that ROS generated from NOX1 in response to LPS may stimulate the impairment of intestinal epithelial TJ formation by up-regulating pro-inflammatory cytokines expression, comprising TNF-α, IL-6, and IL-1β. Indeed, through up-regulation of chemokines, inflammatory cytokines and iNOS, NOX1/NADPH oxidase has a vital role in the TNBS-induced colonic inflammation pathogenesis, according to past studies (Yokota et al., 2017). The Caco-2 cells exposure to IL-1β activated NOX1 expression and ROS generation, leading to increase of epithelial permeability (Tesoriere et al., 2014). In the current study, AB23A attenuated TNF-α, IL-6, and IL-1β expression. AB23A also attenuated ROS generation and significantly reduced NOX1 overexpression in LPS-stimulated Caco-2 monolayers. In addition, using shRNA (to NOX1), we proved that AB23A attenuates ROS generation by inhibiting NOX1 expression in Caco-2 cells. These findings proved that AB23A inhibits the NOX1 expression and subsequent ROS generation in LPS-stimulated Caco-2 monolayers. Moreover, we also found that ZO-1 and occludin expression were elevated after NOX1 knockdown with shRNA (to NOX1) in Caco-2 cells. All these data reveal that NOX1/ROS has a crucial role in intestinal epithelial TJ permeability and AB23A maintains intestinal barrier integrity by suppressing NOX1 expression and reducing the subsequent production of ROS.

Bacterial endotoxin-induced systemic inflammatory response is the main cause of high fat diet-induced metabolic diseases development, including NAFLD or obesity-associated diabetes (Tsalik and Woods, 2009; Porras et al., 2017). As the most important component of bacterial endotoxin, LPS has been shown to aggravate metabolic disorders by elevating IL-1β, TNF-α, and IL-6 expression, which might be related to TLRs (Vaez et al., 2016). TLRs are important proteins involved in non-specific immunity and associated with specific immunity (Wang et al., 2019). TLR4 is a member of the TLR family, as a pattern recognition receptor for LPS from Gram-negative bacteria, which is closely related to immune or inflammatory diseases (Miyake, 2004). In the present study, AB23A inhibits TLR4 overexpression in LPS-stimulated Caco-2 monolayers. By using shRNA against TLR4, we found that ZO-1 and occludin expressions were elevated in Caco-2 monolayers after being stimulated with or without LPS. Moreover, after knocking down TLR4 with shRNA, we found that TLR4 expression influenced NOX1 expression and subsequent ROS production. Consistent with previous studies, TLR can activate NADPH oxidases to produce ROS and modulate inflammation by affecting the expression of NADPH oxidases in different tissues and organs. For example, increased TLR4 signaling in colitis drives DUOX2 expression and H2O2 production in epithelial cells (Burgueño et al., 2021). In ROS-mediated inflammatory diseases, suppressing TLR2 or TLR4 is a novel therapeutic strategy (Hsieh et al., 2016). Wang et al. (2019) indicated that blocking the TLR4/NOX2 signaling might be a potential therapy for endotoxin-induced cardiac dysfunction. Inhibition of NOX1/ROS prevented the enhancement of lung tumor burdens by LPS-induced acute lung infection in non-small cell lung cancer (NSCLC) cells (Liu et al., 2015). Therefore, the TLR4-NOX1/ROS axis may be a potential candidate for AB23A to be used as a protective target to against Intestinal barrier permeability.

In conclusion, despite the importance of our results, the study still has some limitations. In the current study, only Caco-2 monolayers were utilized for investigating AB23A protective effects on intestinal barrier dysfunction and no further validation were conducted *in vivo*. Our findings still demonstrated that AB23A protects the intestinal barrier integrity. AB23A-mediated protective effect mechanisms on intestinal barrier function encompass the up-regulation of TJ-associated proteins and suppression of pro-inflammatory cytokine expression (Figure 7C). The TLR4-NOX1/ROS signaling pathway may be a potential candidate for AB23A to be used as a protective target to against intestinal barrier permeability.

## Data Availability

The datasets presented in this study can be found in online repositories. The names of the repository/repositories and accession number(s) can be found below: https://www.ncbi.nlm.nih.gov/genbank/, NM_001271815.2; https://www.ncbi.nlm.nih.gov/genbank/, NM_003266.4; https://www.ncbi.nlm.nih.gov/genbank/, NM_001101.3.
