# Supplementary material for: Alisol B 23-Acetate Ameliorates Lipopolysaccharide-Induced Intestinal Barrier Dysfunction by Inhibiting TLR4-NOX1/ROS Signaling Pathway in Caco-2 Cells
Source: Front Pharmacol. 2022 Jun 14;13:911196. doi: 10.3389/fphar.2022.911196 (PMC9237229; doi:10.3389/fphar.2022.911196)
Supplement: Supplementary file 1 [file DataSheet1.PDF]

Supplementary Material

1 Supplementary Figure 1

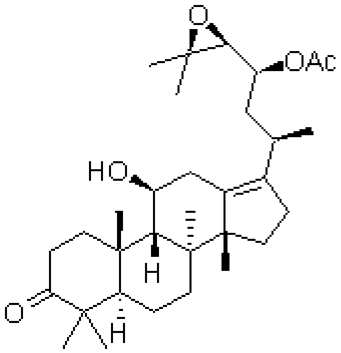

Molecular structure of AB23A.

2 Supplementary Figure 6S

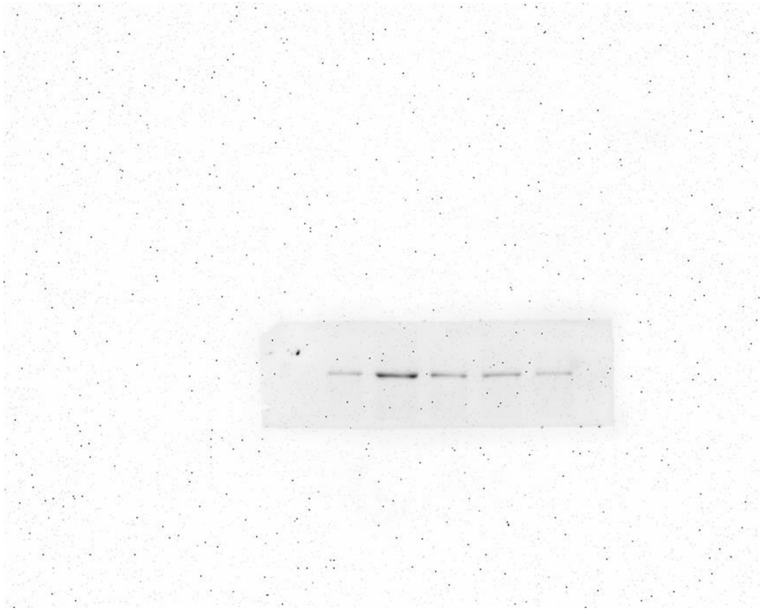

Supplementary Figure 6S1. The full scan of the entire original gel for NOX1.

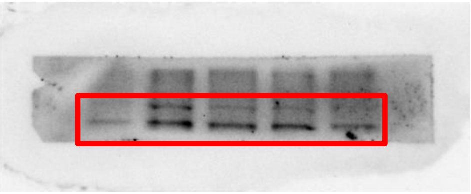

Supplementary Figure 6S2. The full scan of the entire original gel for TLR4.

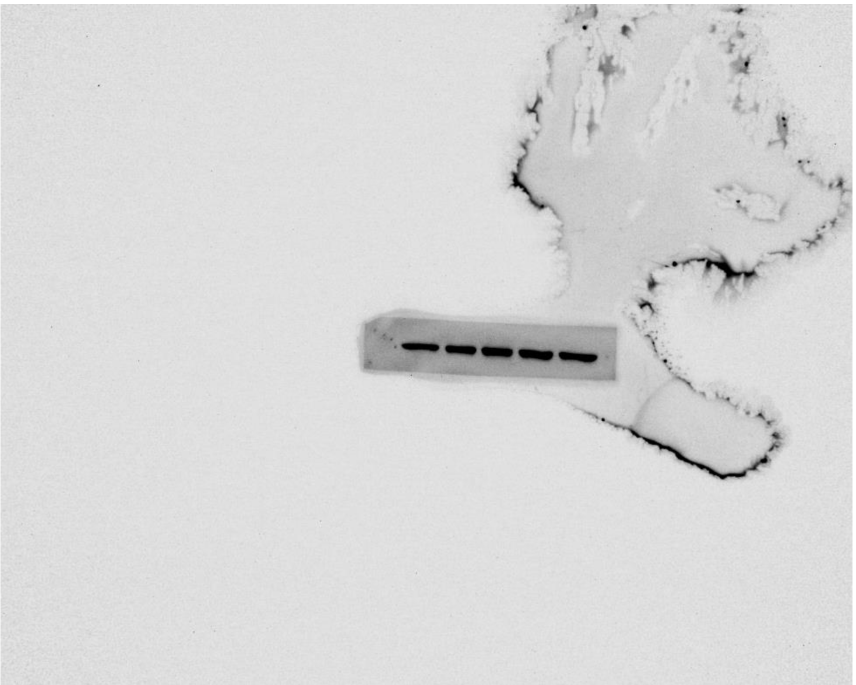

Supplementary Figure 6S3. The full scan of the entire original gel for β-actin in Figure 6 A and B.

3 Supplementary Figure 7S

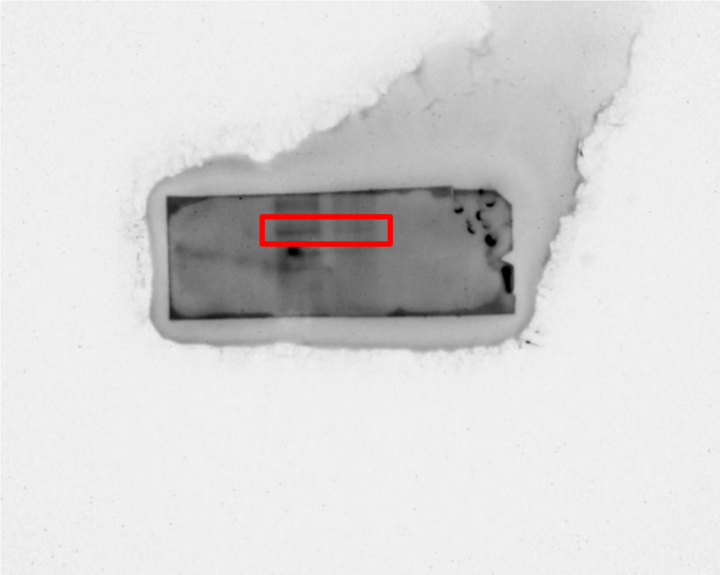

Supplementary Figure 7S1. The full scan of the entire original gel for NOX1.

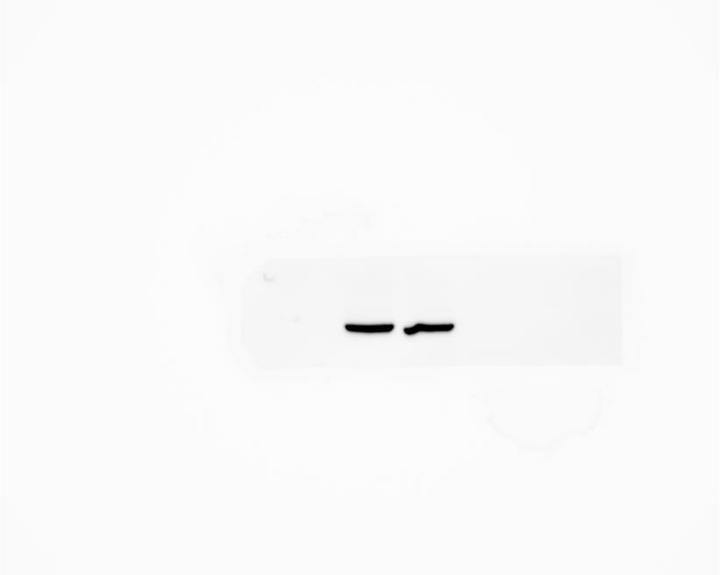

Supplementary Figure 7S2. The full scan of the entire original gel for β-actin.

4 Supplementary Figure 8S

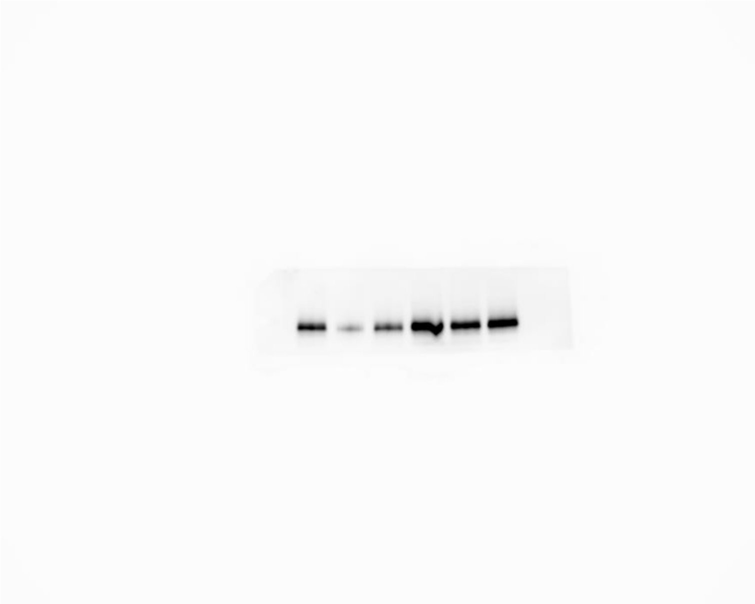

Supplementary Figure 8S1. The full scan of the entire original gel for occludin.

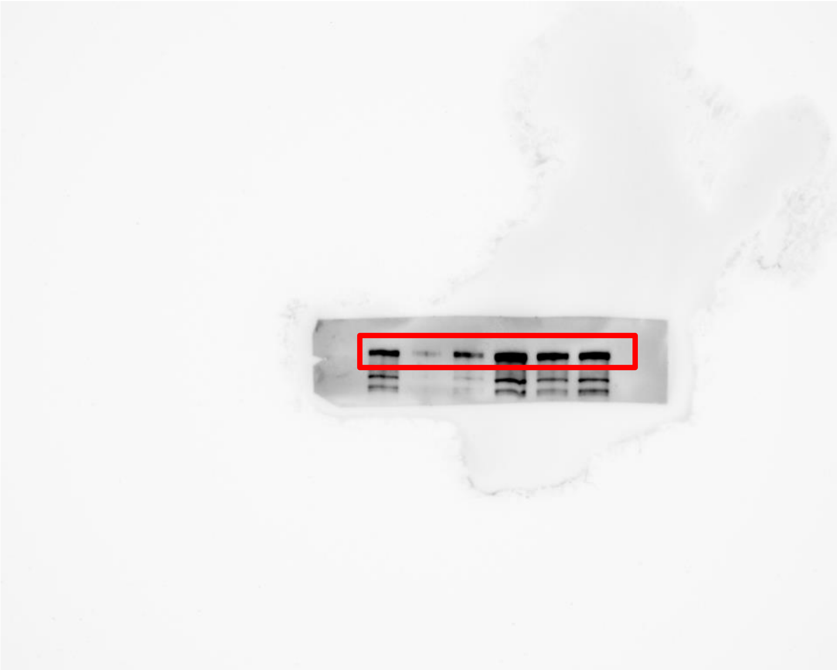

Supplementary Figure 8S2. The full scan of the entire original gel for ZO-1.

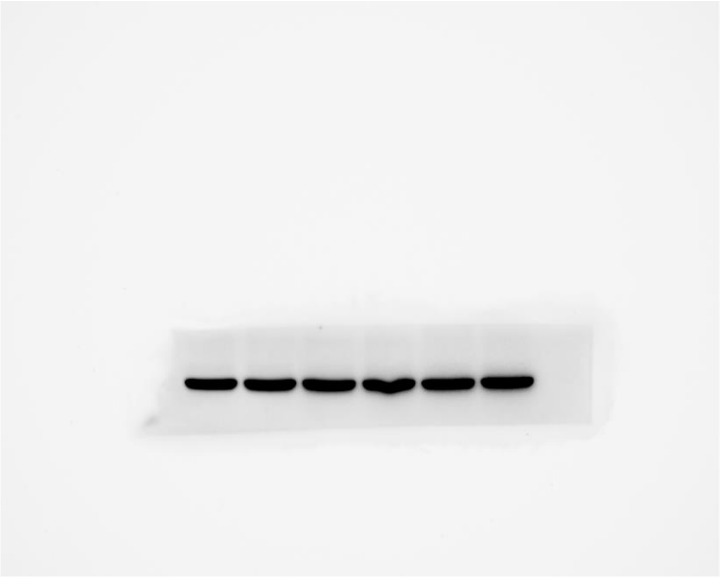

Supplementary Figure 8S3. The full scan of the entire original gel for β-actin in Figure 8A and B.

5 Supplementary Figure 9S

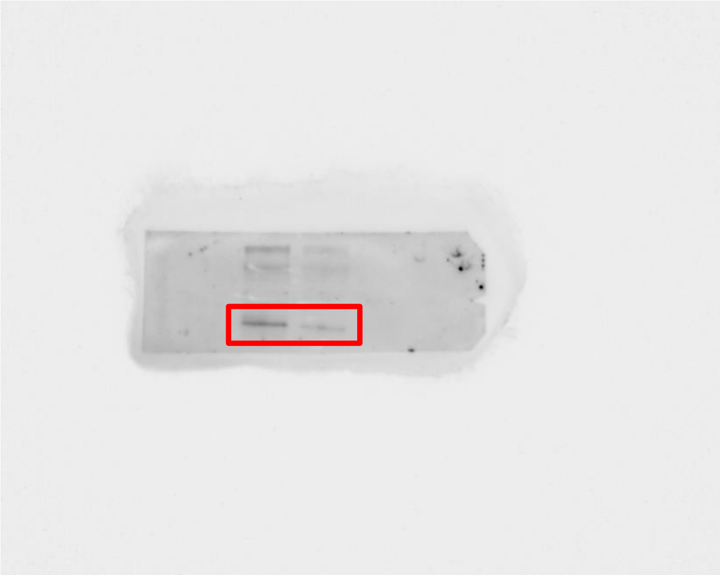

Supplementary Figure 9S1. The full scan of the entire original gel for TLR4 in figure 9B.

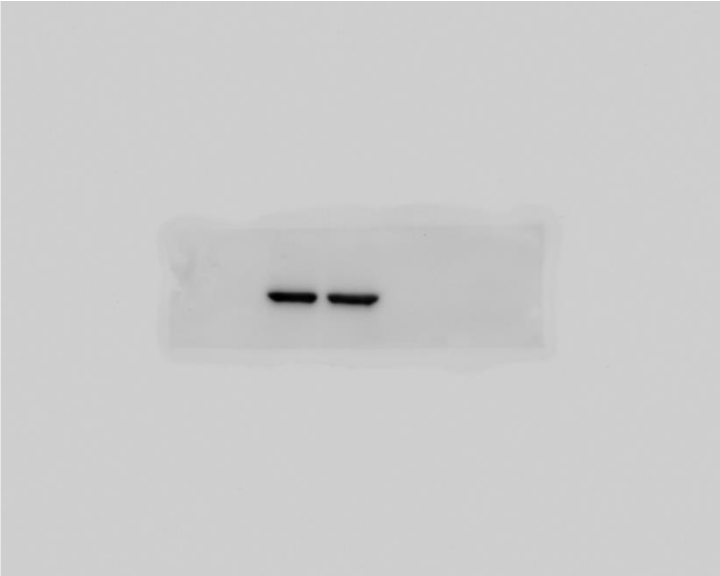

Supplementary Figure 9S2. The full scan of the entire original gel for β-actin in Figure 9B.

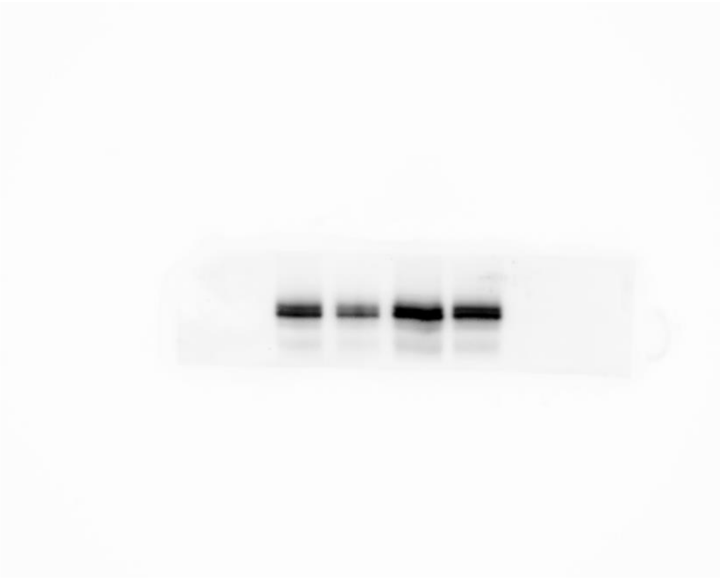

Supplementary Figure 9S3. The full scan of the entire original gel for occludin in Figure 9 C.

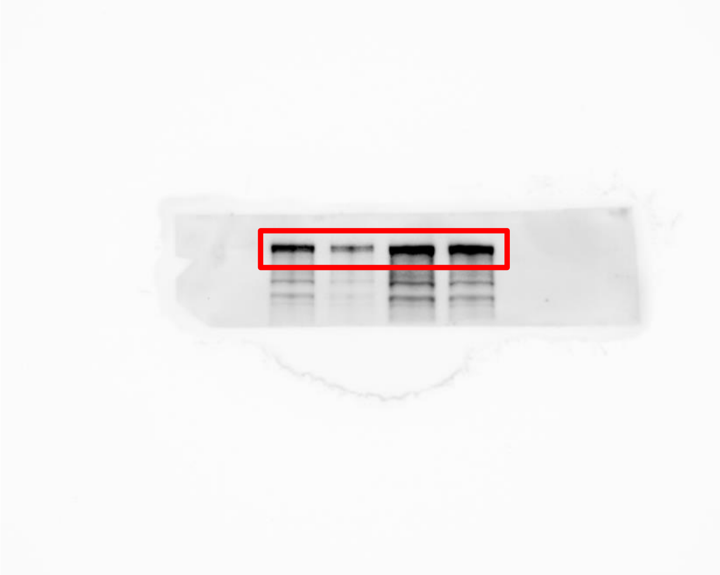

Supplementary Figure 9S4. The full scan of the entire original gel for ZO-1 in Figure 9 D.

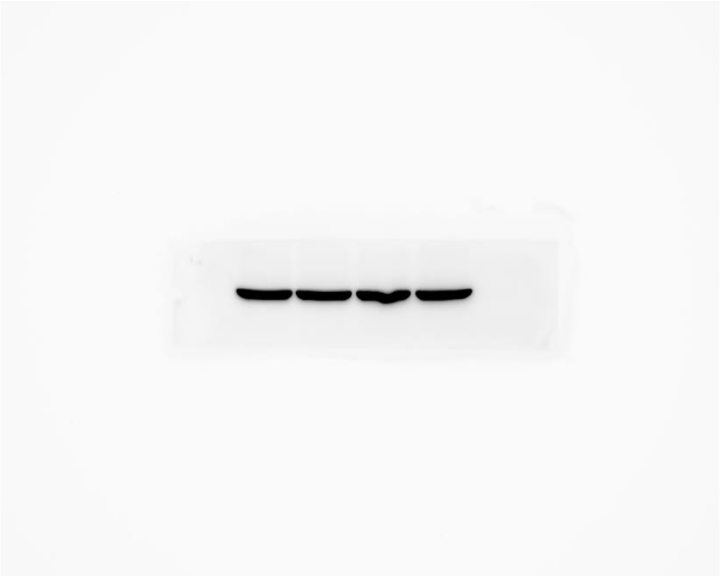

Supplementary Figure 9S5. The full scan of the entire original gel for ZO-1 in Figure 9C and D.

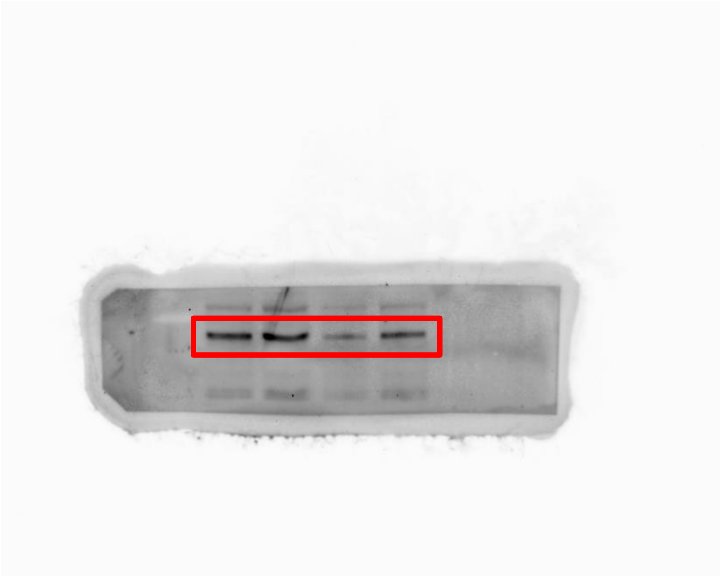

Supplementary Figure 9S6. The full scan of the entire original gel for NOX1 in Figure 9 E.

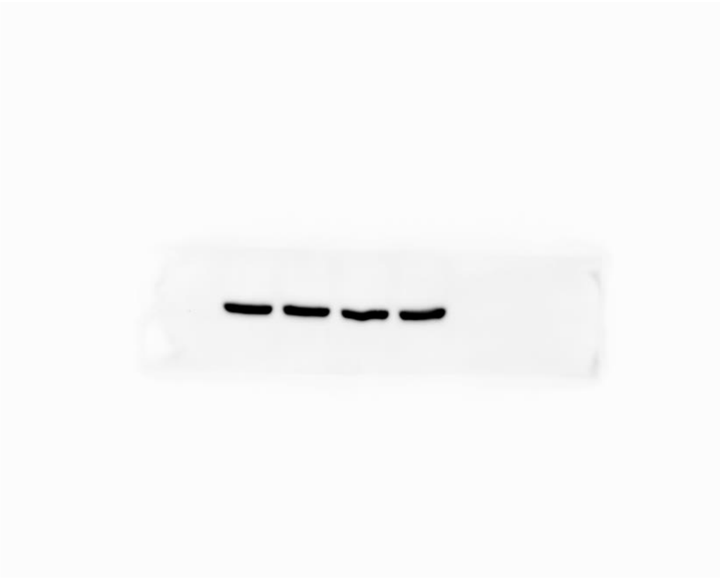

Supplementary Figure 9S7. The full scan of the entire original gel for β-actin in Figure 9 E.
